# Elevated 5hmC levels characterize DNA of the cerebellum in Parkinson’s disease

**DOI:** 10.1038/s41531-017-0007-3

**Published:** 2017-02-01

**Authors:** Reinhard Stöger, Paula J. Scaife, Freya Shephard, Lisa Chakrabarti

**Affiliations:** 1 School of Biosciences, University of Nottingham, Nottingham, LE12 5RD UK; 2 School of Veterinary Medicine and Science, Faculty of Medicine and Health Sciences, University of Nottingham, Nottingham, LE12 5RD UK

## Abstract

5-methylcytosine and the oxidation product 5-hydroxymethylcytosine are two prominent epigenetic variants of the cytosine base in nuclear DNA of mammalian brains. We measured levels of 5-methylcytosine and 5-hydroxymethylcytosine by enzyme-linked immunosorbent assay in DNA from post-mortem cerebella of individuals with Parkinson’s disease and age-matched controls. 5-methylcytosine levels showed no significant differences between Parkinson’s disease and control DNA sample sets. In contrast, median 5-hydroxymethylcytosine levels were almost twice as high (*p* < 0.001) in both male and female Parkinson’s disease individuals compared with controls. The distinct epigenetic profile identified in cerebellar DNA of Parkinson’s disease patients raises the question whether elevated 5-hydroxymethylcytosine levels are a driver or a consequence of Parkinson’s disease.

## Introduction

Parkinson’s disease (PD) is a progressive neurodegenerative disorder with primary impairment of motor control. Functional changes in the cerebellum, a brain structure operative in motor learning and modulation of motor commands, are implicated in the pathophysiology and clinical symptoms of PD.^1^ Within the cerebellum reside two distinctive neuronal cell types, Purkinje cells and granule cells, respectively, along with specialized glial cells, all of which have 5-hydroxymethylcytosine (5hmC) enriched throughout the bodies of expressed genes.^2^ Possible imbalances of 5-methylcytosine (5mC) and 5hmC in cerebellar nuclear DNA of PD individuals have not been investigated before.

## Results and discussion

**Fig. 1:**
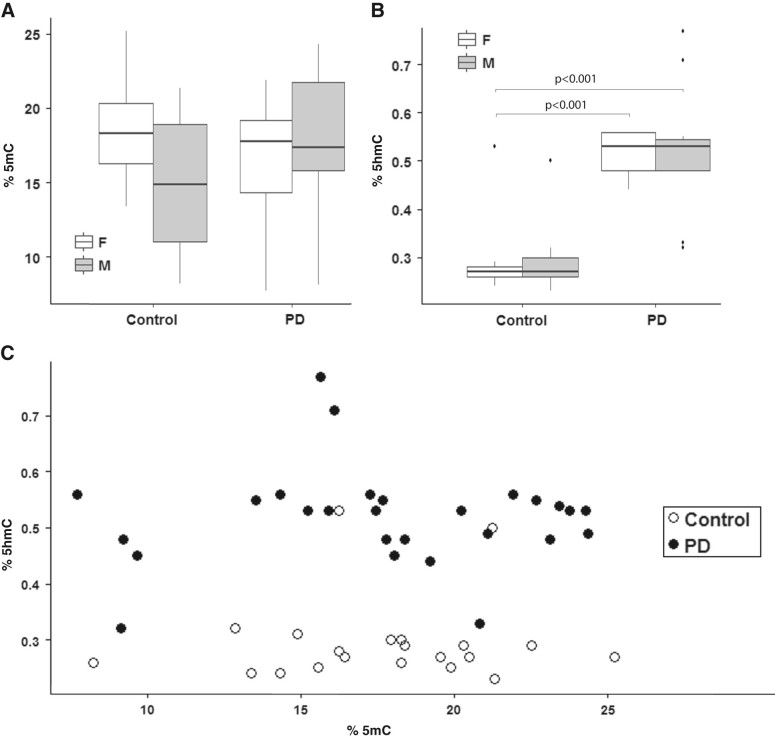
Quantitation of 5mC and 5hmC in DNA of the cerebellum from age-matched control (*n* = 27; 11 females, 16 males) and PD individuals (*n* = 36; 12 females (F), 24 males (M)). Data are presented as box-and-whisker plots and indicate the full range, interquartile range, and median value in each group. *Error bars* represent SEM (**a**, **b**). The *scatter plot* depicts 5hmC and 5mC levels for individuals of the control and PD group (**c**)

Our results demonstrate extensive inter-individual 5mC variation in PD samples (Fig. 1a, c). The broad range of 5mC levels, however, is also manifest in control individuals and does not differ between the two cohorts. Age was considered as a factor contributing to the variability of 5mC levels among individuals. Indeed, PD patients were reported earlier to have an accelerated epigenetic age, a finding that is based on measurements of methylation levels at 353 individual CpG sites in blood DNA.^3^ Quantification of methylation levels at individual CpG sites was not possible with our enzyme-linked immunosorbent assay (ELISA) approach. However, based on our genome-wide methylation measurements—representing the sum total of all cytosine methylation events in cerebellar DNAs—we detected no apparent correlation between 5mC levels and age (Supplementary Fig. 1). This finding is consistent with two previous studies reporting considerable 5mC variation in the cerebellum among individuals who are not affected by Parkinson’s.^4,5^ In comparison, 5hmC levels fall within a narrow range in the PD group, which differs significantly (*p* < 0.001) from the narrow range detected in the control group (Fig. 1b). With the exception of two outliers in the control group, cerebellar DNA samples of PD individuals were found to have higher 5hmC levels (Fig. 1c). The increase in levels of this epigenetic mark is sex-independent and evident in both PD males and PD females (Fig. 1b). Age-related increases in 5hmC levels occur in the mouse cerebellum during the first 12 months of life,^6,7^ a time span representing birth to middle age in humans. The dynamics of cerebellar 5hmC levels in later stages of life are not known to date. We analysed cerebellar DNA samples from PD and control individuals whose age ranged between 58 years and 93 years at time of death. Our data suggest that genome-wide 5hmC levels do not change in the cerebellum as a result of age during this period of life (Supplementary Fig. 1).

Elevated 5hmC levels in PD DNA samples reflect, in part at least, epigenomic changes of granule cells, as these neurons outnumber any other cell type of the cerebellum. It is not clear if the rise of 5hmC is a consequence of an altered physiology of the cerebellum in PD individuals. Compensatory and pathological effects have been proposed to explain the functional changes observed in the cerebellum during the progression of Parkinson’s.^1^ Increased 5hmC levels have been reported before in human post-mortem cerebellum of individuals with autism,^8^ a population group showing high rates of parkinsonism in adulthood.^9^ Our findings motivate an array-based approach to determine the epigenetic age^3^ and, in combination with oxidative bisulfite sequencing, the identification of genomic regions and genes that show an altered abundance of 5hmC in the cerebellum.^5,10^ Maps and measurements of 5hmC in different anatomical brain regions will reveal possible common molecular pathology shared by conditions manifesting as parkinsonism.

## Materials and methods

### Cerebellum samples

Human brain sections and frozen brain samples were obtained from the Human Tissue Authority-approved, Nottingham Health Science Biobank (Nottingham University Hospitals NHS Trust), and Parkinson’s UK Brain Bank (Imperial College London). The tissue banks granted us use of the tissue as end users. The Parkinson’s tissue bank has approval from the Research Ethics Committee for Wales ref 08/MRE09/31+5. The tissue collection and procedures at the Nottingham University Hospitals Biobank have been ethically approved by the Greater Manchester National Research Ethics Service. This study was granted specific ethics approval by both of the ethics committees serving the biobanks and also by the local ethics committee at the School of Veterinary Medicine and Science at the University of Nottingham.

Age at death for PD individuals ranged between 63–87 years (females) and 58–83 years (males); for non-PD (control) individuals age at death ranged between 61–93 years (females) and 58–90 years (males). For each PD sample used in this study, the Biobanks provided us with age-matched control samples, which differed +/−5 years of age, or less. We performed analysis of covariance to explore whether covariates such as post mortem interval or a different tissue collection centre could have influenced our results. We detected no effects of confounding variables (Supplementary Table 1).

### Nuclear DNA extraction

Nuclear DNA pellets were isolated from human cerebellar tissue as previously described^11^ and resuspended in 500 μl TE buffer (pH 7.5). Samples were re-pelleted (13,000×*g* for 5 min) before being resuspended in 375 µl TE buffer with 20 µl lysis buffer (20% w/v SDS in 50 mM Tris, 20 mM EDTA pH 7.8) and 3 µl Proteinase K (20 mg/ml) and were incubated at 37 °C for 1 h. In order to precipitate protein cell wall debris, 200 µl saturated NaCl (>6.0 M) was added, mixed thoroughly by inversion and centrifuged at 12,500×*g* for 10 min. The supernatant was transferred to a fresh tube and an equal volume of Tris-equilibrated phenol:chloroform:isoamyl alcohol (25:24:1, Sigma Aldrich, Poole, UK) was added. This was briefly agitated and centrifuged at 13,000×*g* for 3 min. The upper aqueous phase was transferred to a fresh tube. DNA was precipitated by adding 30 µl 1 M Na acetate (pH 5.2) and two volumes pre-chilled absolute ethanol and incubating at 4 °C for 2 h. Precipitated DNA was centrifuged at 13,000×*g* for 20 min. DNA was then washed with cold 70% ethanol, thoroughly air-dried and resuspended in TE buffer containing 20 µg/ml RNaseA. A Qubit dsDNA BR assay (Life Technologies DNA, Warrington, UK) was used to quantify DNA. DNA quality was determined using a Nanodrop 8000 spectrophotometer (Thermo Fisher Scientific, Loughborough, UK).

### Quantification of global DNA methylation and hydroxymethylation

Cerebellar genomic 5mC and 5hmC levels of PD and controls were determined by colorimetric ELISA using the 5mC DNA ELISA kit and Quest 5hmC DNA ELISA kit, respectively (Zymo Research, Irvine, CA, USA). PD and control DNAs were analysed simultaneously and different batches of ELISA kits were used to analyse all DNA samples, thereby limiting variation in measurements resulting from potential procedural or batch discrepancies. Assays were performed according to the manufacturer’s instructions loading 100 ng of DNA per well. Absorbance at 405 nm was captured using a LT4000 microplate reader (LabTech International, East Sussex, UK). The percentage of global 5mC and 5hmC is expressed as mean ± standard error mean (SEM).

### Statistical analysis

All analyses were performed using IBM SPPS Statistics version 22 (IBM, Leeds, UK). The Kolmogorov–Smirnov test was used to assess the distribution of data. Depending on distribution of data, between-group comparisons were made using either one-way analysis of variance or the Kruskal–Wallis test as appropriate, and if significant were followed by a Bonferroni post hoc test or Mann–Whitney U test, respectively. Values of *p* ≤ 0.05 are considered significant. Unless otherwise stated all data are expressed as mean (standard error of mean).

## Electronic supplementary material

Supplementary Information
