# Supplementary material for: Elevated 5hmC levels characterize DNA of the cerebellum in Parkinson’s disease
Source: NPJ Parkinsons Dis. 2017 Feb 1;3:6. doi: 10.1038/s41531-017-0007-3 (PMC5460211; doi:10.1038/s41531-017-0007-3)
Supplement: Supplementary file 1 — Supplementary Information [file 41531_2017_7_MOESM1_ESM.pdf]

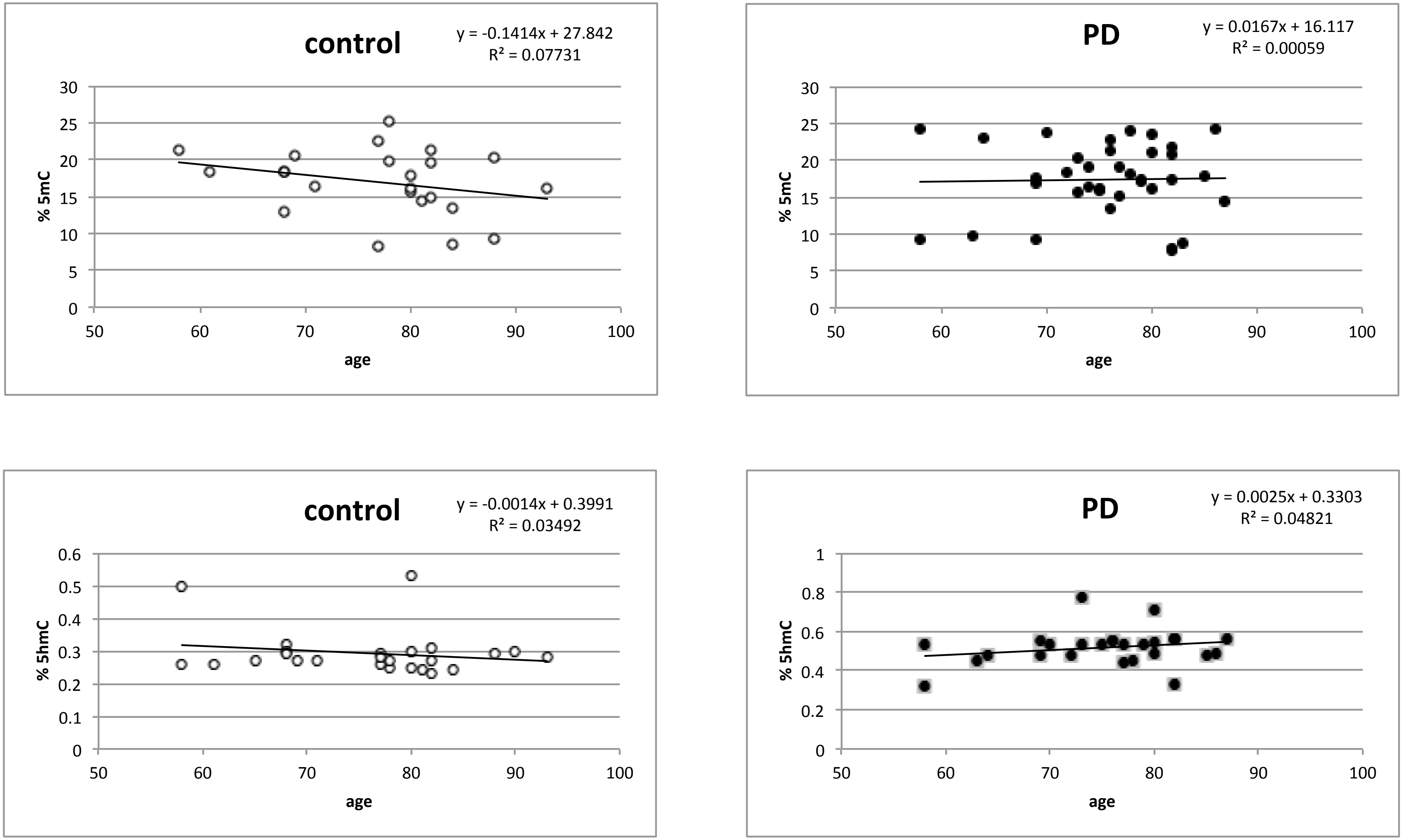

Supplemental figure 1:
Genome-wide 5mC and 5hmC levels in cerebellar DNA of PD and age-matched control individuals in relation to age.

Stöger et al. *Elevated 5hmC levels characterize DNA of the cerebellum in Parkinson's disease*
npj Parkinson's Disease

	Unadjusted mean	Adjusted mean for time to postmortem	Adjusted mean for collection centre
Control	0.2932	0.293	0.293
PD	0.5178	0.518	0.52

Supplemental table 1:
We performed analysis of covariance (ANCOVA) to explore whether covariates such as post mortem interval and different tissue collection centre could have influenced our results. We detected no effects of confounding variables; the table gives an estimation of the means adjusted for either of the covariates as shown above.

Stöger et al. *Elevated 5hmC levels characterize DNA of the cerebellum in Parkinson's disease*
